# Behavior of Mold Electromagnetic Stirring for Round Bloom Castings and Its Eccentric Stirring Problem

**DOI:** 10.3390/ma15248814

**Published:** 2022-12-09

**Authors:** Pu Wang, Hong Xiao, Zhuang Zhang, Shaoxiang Li, Jiaquan Zhang

**Affiliations:** 1 School of Metallurgical and Ecological Engineering, University of Science and Technology Beijing, Beijing 100083, China; 2 Magnetoelectric Research Institute, Hunan Zhongke Electric Co., Ltd., Yueyang 414000, China; 3 School of Materials Science and Engineering, Tsinghua University, Beijing 100083, China

**Keywords:** round bloom, electromagnetic torque, optimal stirring frequency, copper tube magnetic shielding, eccentric stirring

## Abstract

In this paper, a mold electromagnetic stirring (M-EMS) model was established to investigate the behavior of M-EMS for round bloom castings under different conditions, and an electromagnetic-flow-heat transfer-solidification coupling model was established to explore the problem of eccentric stirring for various formats of round blooms. The results show that the magnetic flux density decreased with the increase in the current frequency, but the electromagnetic torque increases first and then decreases with it, and the same structure of M-EMS for round blooms has the same optimal current frequency at any current intensity. The electromagnetic torque and electromagnetic force both increase as a quadratic function of the current intensity, and the electromagnetic torque, which drives the steel flow, can directly characterize the real M-EMS performance. The mold copper tube has a significant magnetic shielding effect on M-EMS, the stirring intensity decreases rapidly as the tube thickness increases, and the optimal stirring frequency decreases as well. In fact, the deviation between the stirrer center and the geometric center of the strand can result in the eccentric stirring phenomenon. When blooms with a section size of Φ350 mm are produced by Φ650 mm SMS-Concast casting machine, the upper region of the inner arc side and the lower region of the outer arc side are subject to a stronger washing effect, which makes the temperature of the inner and outer arcs show alternating differences. The jet flow from the five-port nozzle can suppress the difference in initial solidification symmetry between the inner and outer arcs of round blooms caused by eccentric stirring. This paper reveals the magnetic shielding effect of the mold copper tube and the magnetic field loss of the air between the stirrer and the inner and outer arcs of the copper, which lead to the stirring intensity and the eccentric stirring phenomenon.

## 1. Introduction

With the development of round bloom continuous casting (CC) technology and the expansion of the application range of round blooms, an important feature of the development of round bloom CC in recent years is to increase the arc radius of the CC machine and expand the casting size. The diameter of large-section round bloom has been increased from Φ400 mm to Φ750 mm, and even Φ1000 mm above; correspondingly, the radius of the machine arc is also required to increase from 14 m to 16.5 m, with the current maximum of 17.5 m or above [1,2,3]. However, due to the characteristics of round bloom CC, such as large section, low casting speed, and poor feeding ability of molten steel at the solidification end, serious defects such as composition segregation, central porosity and shrinkage cavity are prone to occur in the CC process [4,5,6]. Among them, once macro-segregation defects formed, it was difficult to eliminate them through subsequent steps such as heating, rolling, heat treatment and forging. At present, mold electromagnetic stirring (M-EMS) is the basic method for comprehensive quality control of special steel round blooms [7,8,9]. However, the magnetic field acting in the strand is attenuated due to the magnetic shielding of the mold copper tube, and obtaining sufficient stirring intensity in the round bloom CC is still a challenge for M-EMS equipment and process design.

Under the action of M-EMS, the molten steel can effectively scour and wash the solidification front, inhibit the initial solidification shell from capturing bubbles and inclusions, and effectively reduce the porosity and content of inclusions on the surface of the strand [10,11,12]. M-EMS can make the molten steel in the mold flow sufficiently and keep the molten steel temperature uniform; then the uniform shell thickness can reduce the degree of surface cracks and even reduce the risk of breakout, which greatly improves the surface quality of the strand [13]. In addition, Wu and Harada [14,15] found that the dendrite spacing decreased significantly with the increase in the electromagnetic stirring intensity, the grains were refined when the equiaxed region was significantly enlarged, and the central segregation was also significantly improved. A large number of studies have shown that the current intensity and current frequency of M-EMS have a great impact on the magnetic field. The magnetic flux density increases with the increase in current intensity, but decreases with the increase in current frequency [16,17,18,19,20,21]. However, the magnetic flux density is only an intermediate parameter between the excitation current of the stirrer and the electromagnetic force induced in the molten steel, and cannot directly reflect the stirring intensity. Sha [22] carried out a numerical simulation study on the horizontal CC process of round copper billet with electromagnetic stirring, and found that even though the magnetic flux density decreases with increasing current frequency, the electromagnetic force increases with increasing current frequency. Ren [23] found that even without the mold copper tube, the electromagnetic force at the R/2 position inside the round bloom will first increase and then decrease with the increasing current frequency of the stirrer, and a peak in the electromagnetic force will exist under the magnetic shielding of the strand. Geng [24] revealed the existence of an optimal stirring frequency for M-EMS corresponding to the maximum electromagnetic force using numerical simulations, below which the tangential flow velocity of the molten steel at the meniscus increases with increasing frequency. Yu and Zhu [25] proposed, by numerical simulation prediction and industrial test demonstration, that the stirring intensity increases with the increase in current frequency or current intensity when the current frequency is lower than the optimal value, which can effectively enhance the effect of M-EMS to optimize the strand quality. Fang [26] found that with the downward shift of the M-EMS installation position, the degree of liquid level fluctuation was reduced, the shell thickness at the mold exit increased, and the subsurface negative segregation increased, suggesting that the content of non-metallic inclusions in the strand can be reduced by a moderate installation position. The electromagnetic force cannot be measured due to the high temperature of molten steel in actual CC production. An [27] and Li [28] both designed electromagnetic torque measurement devices to obtain the induced electromagnetic torque in the strand by experimental measurement and model calculation. In addition, when M-EMS was installed at the position not too far beyond the mold, an optimal stirring frequency corresponding to the maximum stirring intensity of M-EMS was proven to exist.

The actual large-section round bloom casting machine often produces strands with different cross-section specifications. When producing smaller-section products, the phenomenon of deviation between the M-EMS center and the geometric center of the strand will occur, but how the inconsistency between copper tube magnetic shielding and the loss of magnetic field in the air affects the real stirring performance of M-EMS is rarely studied. On this basis, an electromagnetic model was established to explore the characteristics of M-EMS of round blooms under different conditions, and an empirical formula theoretical analysis and electromagnetic torque model were combined as the basis for determining the optimal stirring frequency of M-EMS under the magnetic shielding effect of copper tubes. This paper reveals the magnetic shielding effect of the mold copper tube, and the magnetic field loss of the air between the stirrer and the inner and outer arcs of the copper, which lead to the stirring intensity and the eccentric stirring phenomenon. At the same time, a coupling model of electromagnetic, flow, heat transfer and solidification were established to explore the eccentric stirring problem of the round bloom, in order to provide theoretical guidance for the optimization of the M-EMS in the actual round bloom CC to control the strand quality.

## 2. Mathematical Model

**Figure 1:**
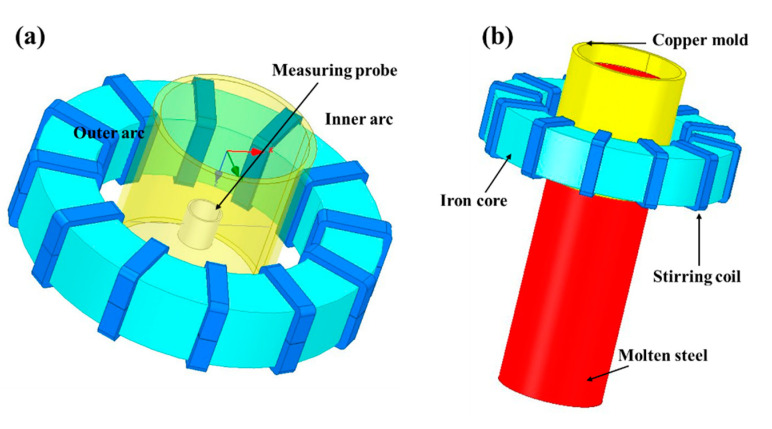
Schematic diagram of electromagnetic model: (**a**) aluminum probe load; (**b**) molten steel load.

**Figure 2:**
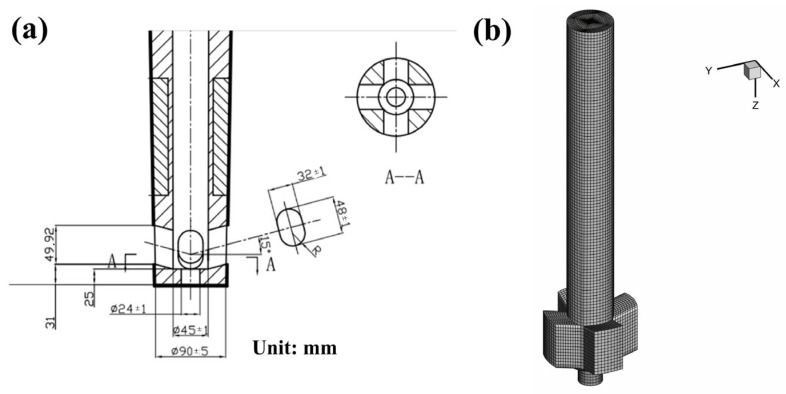
Schematic of the five-port nozzle: (**a**) geometry; (**b**) mesh.

Based on the R14 m arc round bloom casting machine of SMS-Concast for the production of 42CrMo gear steel, Figure 1 shows the schematic structure of the M-EMS model for the round bloom. The electromagnetic model includes the core, twelve rows of coils with three pairs of windings, the mold copper tube, the strand, the aluminum probe (with the conductivity of 3.8 × 10^7^ S·m^−1^) and the enclosed air domain (not shown). The magnetic flux density and torque without molten steel load were analyzed, and the electromagnetic force information with molten steel load was extracted. The stirrer has an inner diameter of 1010 mm, an outer diameter of 1510 mm, and a height of 300 mm. The stirring method is continuous horizontal rotation. The origin of the coordinates in the Cartesian coordinate system is located at the center of the meniscus, the Z-direction is the casting direction, the stirrer center is Z = 400 mm, and the X and Y-directions are the radial direction of the strand parallel to the centerline of the side ports. Among them, combining with the effect of the nozzle in the actual production of round bloom CC in this plant, a five-port nozzle is selected for the fixed injection method [29]. The upper edge of the side port is 120 mm from the meniscus, and its geometry and mesh are shown in Figure 2.

### 2.1. Coupling Model

Table 1 summarizes the governing equations of the electromagnetic, flow, heat transfer and solidification coupling model. The following basic assumptions are made to reasonably describe the complex and interacting metallurgical physical phenomena in the mold:

(1) Ignoring the effects of mold taper, mold vibration, strand curvature, and solidification shrinkage on the flow and temperature state of the internal molten steel, the casting process is steady state with constant casting speed, superheat degree, and cooling conditions.
(2) The molten steel is considered as an incompressible Newtonian fluid and the thermal parameters such as density, viscosity, specific heat and thermal conductivity are treated as constants.
(3) The position of the solidification front in the casting process is variable, so it is difficult to apply the wall function to the standard k−ε model. The mushy zone is considered as a porous media region, and the turbulence effect is calculated by the low Reynolds k−ε model [30,31].
(4) The magnetic Reynolds number R_m_ is much less than 1 in the mold metallurgical process, i.e., the influence of the molten steel flow on the magnetic field is negligible.
(5) Under the low-frequency condition of M-EMS, the period of the alternating current controlling the change of magnetic field is much smaller than the momentum response time of the molten steel, so the time-varying electromagnetic force can be replaced by the time-averaged electromagnetic force under the harmonic magnetic field [32].
(6) The Joule heat from M-EMS and the heat from the solid-state phase change after solidification are much smaller than the latent heat of solidification, and their effects on heat transfer and solidification are ignored.

### 2.2. Boundary Conditions

#### 2.2.1. Electromagnetic Field

1. Three pairs of coil windings are loaded with a three-phase alternating current; the phase difference of each phase is 120°.
2. The magnetic flux lines are parallel to the boundary at the external surfaces of the surrounding air unit.
3. Insulation boundary conditions are imposed between the electromagnetic stirrer coil and the iron core.

#### 2.2.2. Flow and Solidification

Inlet of nozzle: the inlet velocity is derived from the mass conservation relationship of the bloom casting. The inlet temperature is the casting temperature, and the turbulent kinetic energy and turbulent dissipation rate at the inlet are calculated according to the empirical formulas:

(1) $${k=0.01v_{\mathrm{in}}}^{2}$$

(2) $$\varepsilon =2\times \frac{k^{1.5}}{D}$$

where $v_{\mathrm{in}}$ is the velocity of the nozzle inlet and D is the diameter of the nozzle.

Outlet of computation domain: the fully developed flow condition is adopted, where the normal gradients of all variables are set to 0.

Free surface: Considering the insulation effect of the mold flux, the free surface is set to be adiabatic and the shear stress is 0.

Wall surface: a non-slip wall surface is adopted and the nozzle wall is set to adiabatic. The heat flux boundary condition is applied on the mold zone according to:

(3) $$q_{s}=2^{}680^{}000-b\sqrt{\frac{L}{v_{c}}}$$

where $q_{s}$ is the heat flux density. The convective heat transfer boundary condition is imposed on the secondary cooling zone. The integrated heat transfer coefficient is calculated by:

(Water spray region)

(4) $$h_{1}=420w_{0}{}^{0.351}$$

(Air-mist spray region)

(5) $$h_{2}=116+10.44w_{1}{}^{0.851}$$

where $w_{0}$ and $w_{1}$ are the cooling water volume flow rate with a unit of L·m^−2^·s^−1^ and L·m^−2^·min^−1^, respectively.

### 2.3. Solver Setting

The electromagnetic field is calculated by the finite element software Ansys Maxwell (ANSYS, Inc., Canonsburg, PA, USA), and the time-average electromagnetic force is extracted by the coordinate interpolation algorithm. The fluid flow, heat transfer, and solidification of molten steel in the continuous casting process are simulated by finite volume software ANSYS Fluent16.0 (ANSYS, Inc., Canonsburg, PA, USA). In order to fully develop the turbulence in the coupling model, the calculation domain is extended to 2.632 m. The electromagnetic calculation was performed using a tetrahedral grid with a grid number of 418,230. The fluid calculation was performed with a hexahedral mesh, and the encryption operation was applied in the intense transmission and the solidification region, and then the mesh number was 3.9 million. The SIMPLE scheme was used for iterative calculation, and the convergence criterion was set below 10^−6^ for the energy equation and below 10^−4^ for other equations. The structure, material and electromagnetic parameters involved in the model are shown in Table 2, and R_0_ is defined as the dimensionless number of the radius:

(6) $$R_{0}=\frac{r}{R}$$

where, r is the X coordinate in the radial direction and R is the radius for any section.

### 2.4. Numerical Model Validation

**Figure 3:**
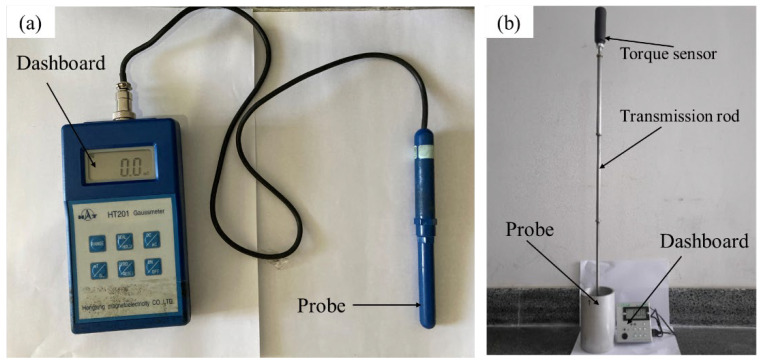
Magnetic field measurement instruments: (**a**) HT-201 Gaussmeter, (**b**) CEDARDIS-RL electromagnetic torque meter.

The electromagnetic characteristics judged at room temperature are different from the real situation [34,35], so the magnetic field measurement was performed when the copper tube was at online temperature during multiple periodic stages at the interval between two castings. The HT-201 Gauss meter for measuring magnetic flux density and the CEDARDIS-RL electromagnetic torque meter for measuring torque values are shown in Figure 3. The aluminum probe with good electrical conductivity is rotated by the electromagnetic torque of the magnetic field and transmitted through the torque transmission rod. The sensor receives the displacement signal and converts it into the electrical signal, then the real-time electromagnetic torque is displayed on the meter.

**Figure 4:**
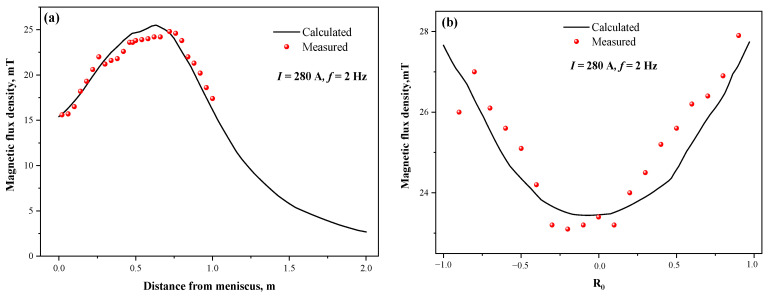
Comparison of calculated and measured values of magnetic flux density: (**a**) vertical center line; (**b**) horizontal radial line.

Figure 4 displays the measured and calculated values of magnetic flux density on the vertical center line and horizontal radial line of the stirrer. It can be seen that the magnetic flux density on the vertical center line increases with the distance from the meniscus and then decreases, showing a distribution trend of “large in the middle and small at both ends”, while the horizontal radial direction is “small in the middle and large at both ends”. The scatter points of each measured value coincide well with the curves of the calculated values, indicating good reliability of the electromagnetic model for the round bloom in this study. In addition, the reliability of the coupled flow-heat transfer-solidification model has been demonstrated through several experiments [34,36,37].

**Figure 5:**
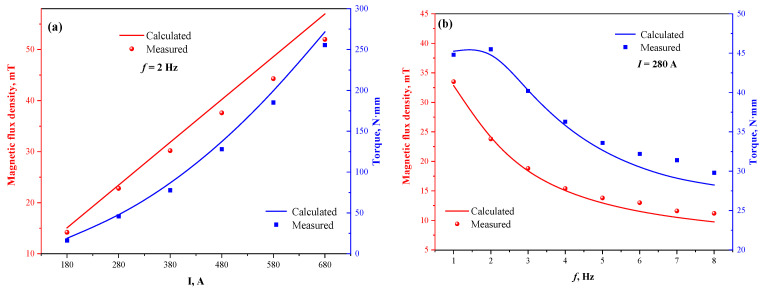
Comparison between measured and calculated values of magnetic flux density and probe torque at the center point of the stirrer: (**a**) different current intensities; (**b**) different current frequency.

The magnetic flux density and probe torque values at the center point of the stirrer were calculated and measured at different current intensities and frequencies, respectively, and plotted as shown in Figure 5. As can be seen from the figure, when increasing the current intensity at a fixed current frequency, the magnetic flux density at the center of the stirrer increases linearly, while the probe torque value increases quadratically. At a fixed current intensity, the magnetic flux density at the center point decays rapidly with increasing current frequency and then decreases slowly, while the probe torque value has a maximum value near 2 Hz. Each measured value is roughly consistent with the calculated value, which can further indicate the high accuracy of the M-EMS model. The measured value is gradually smaller than the calculated value as the current intensity is increased because the magnetic saturation characteristics of the silicon steel core are not considered in the model, and the measured value is gradually larger than the calculated value as the current frequency is increased because the Joule heating effect of the mold copper tube is not considered in the model, which is consistent with the research of Li [28]. In addition, the opposite variation pattern of magnetic flux density and torque values in the range of 1 Hz to 2 Hz indicates that the magnetic flux density as an intermediate parameter between current and electromagnetic force cannot accurately reflect the strength of the stirrer driving the molten steel flow, and the electromagnetic force cannot be measured in the presence of the steel during the actual production process, as the electromagnetic torque driving the molten steel flow can directly characterize the real electromagnetic stirring performance. Meanwhile, according to the research by Raj et al. [38], the actual stirring intensity of M-EMS at low frequency is described by the empirical equation:

(7) $$F_{M}\propto B^{2}f$$

The derivative with respect to f yields:

(8) $$\frac{\partial F_{M}}{\partial f}\propto B^{2}+2B\frac{\partial B}{\partial f}f$$

where the magnetic flux density B decreases with the increase in current frequency, so $\frac{\partial B}{\partial f}$ < 0. When $f\in (0,-\frac{B}{2}/\frac{\partial B}{\partial f})$, the stirring intensity is positively correlated with the current frequency; when the frequency continues to increase, the stirring intensity is negatively correlated with f. Therefore, the feasibility of characterizing stirring intensity by electromagnetic torque is confirmed by combining experimental measurement and theoretical analysis.

## 3. Behavior of M-EMS for Round Blooms

### 3.1. Effect of Current Parameters

**Figure 6:**
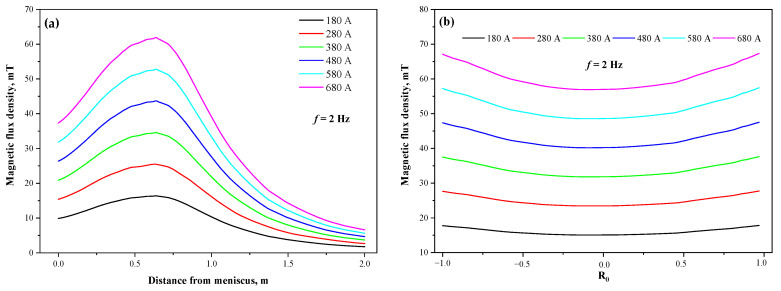
Distribution of magnetic flux density at different current intensities: (**a**) vertical center line; (**b**) horizontal radial line.

Figure 6 illustrates the distribution of magnetic flux density on the central axis and the horizontal radial line of the stirrer at different current intensities for M-EMS, when the section is Φ650 mm and the current frequency is 2 Hz. It can be seen that with the increase in current intensity, the magnetic flux density at the same position in axial and radial directions increase significantly, and the maximum values on the central axis are all located at the same location.

**Figure 7:**
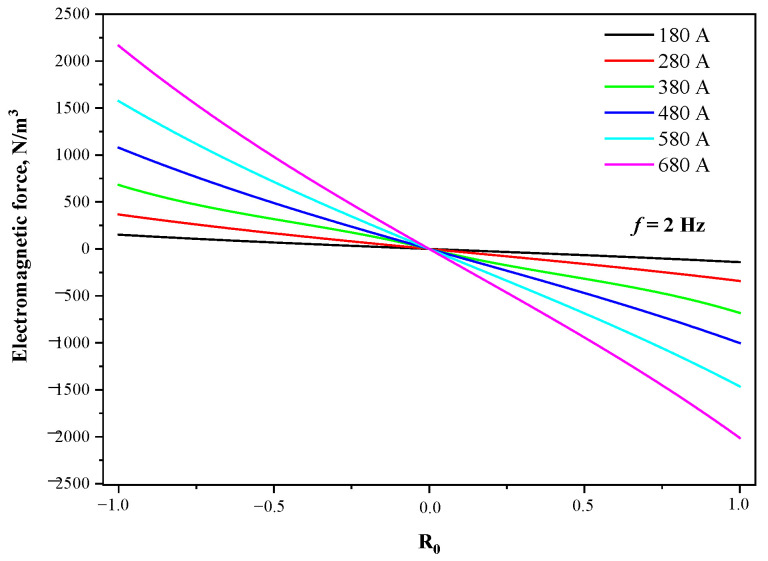
Distribution of electromagnetic forces on radial lines at different current intensities.

Figure 7 compares the electromagnetic force at different current intensities in the radial line of the stirrer center, when the section is Φ650 mm and the current frequency is 2 Hz. The positive or negative values represent the direction of the electromagnetic force. The electromagnetic force increases rapidly with the distance from the center, and the maximum value is located at the edge of the strand. When the current intensity is 180 A, 280 A, 380 A, 480 A, 580 A, and 680 A, the maximum electromagnetic force is 151 N·m^−3^, 367 N·m^−3^, 681 N·m^−3^, 1078 N·m^−3^, 1574 N·m^−3^, and 2163 N·m^−3^, respectively, and the growth rate is gradually accelerating.

**Figure 8:**
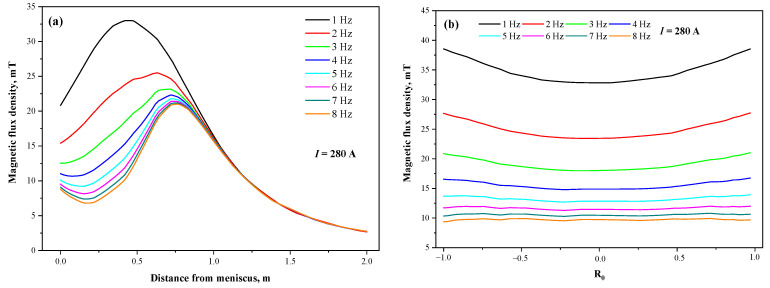
Magnetic flux density distribution at different current frequencies: (**a**) vertical center line; (**b**) horizontal radial line.

Figure 8 indicates the distribution of magnetic flux density on the center axis and horizontal radial line of the stirrer at the different current frequencies when the section is Φ650 mm and the current intensity is 280 A. In fact, according to Abe’s law, the current frequency does not affect the magnetic flux density, and the magnetic shielding effect is quite obvious at the copper tube thickness of 31 mm, so the magnetic flux density at the same location decreases with the increase in current frequency. The position of the maximum magnetic flux density on the central axis also gradually shifts downward with increasing frequency until it stabilizes at the mold exit. The magnetic field at the frequency of 8 Hz can only effectively penetrate a copper tube with a thickness of 31.5 mm under the skin effect of the electromagnetic field, and the magnetic flux density near the meniscus decreases with increasing distance from the meniscus at a higher frequency, which is related to the equivalent penetration thickness of the magnetic field reaching this location. The magnetic shielding effect after exiting the mold copper tube is substantially weakened, and finally the magnetic flux density curves at each frequency basically overlap.

**Figure 9:**
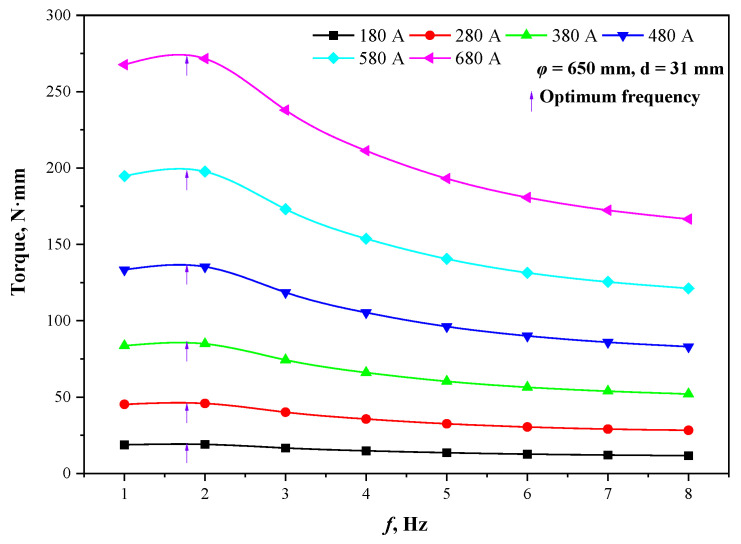
Torque at different current parameters.

Figure 9 shows the torque at the different current frequencies when the section is Φ650 mm and the thickness of the copper tube is 31 mm. The torque values at any current intensity increase and then decrease with increasing frequency, and the maximum values of torque are located at the same frequency (1.8 Hz), indicating that the optimal frequency for the stirrer with a fixed structure is independent of the current intensity. Meanwhile, the torque values increase quadratically with the increase in current intensity at any frequency, which is consistent with the aforementioned growth law of electromagnetic force, and conforms to the variation law of theoretical stirring intensity with current intensity, further confirming the feasibility of torque reflecting stirring capacity.

### 3.2. Influence of Copper Tube Thickness and Round Bloom Section

**Figure 10:**
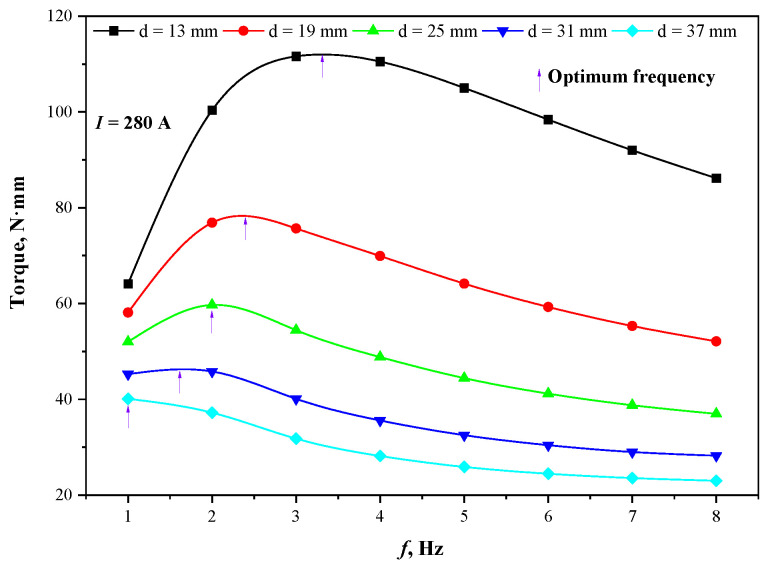
Effect of different thickness of mold copper tube on stirring intensity.

The actual working capability of the stirrer is determined by the penetration depth of the magnetic field, and different thicknesses of mold copper tube are designed to explore the influence on the optimal frequency, as shown in Figure 10. When the copper tube thickness is increased from 13 mm to 37 mm, the optimal frequency of the stirrer decreases from 3.3 Hz to 1.0 Hz, and the stirring intensity decreases rapidly. Consideration can be given to reducing the thickness of the copper tube with sufficient mechanical and thermal strength to alleviate the shielding effect on the magnetic field, to improve the conversion efficiency between the electrical energy and kinetic energy of the stirrer.

**Figure 11:**
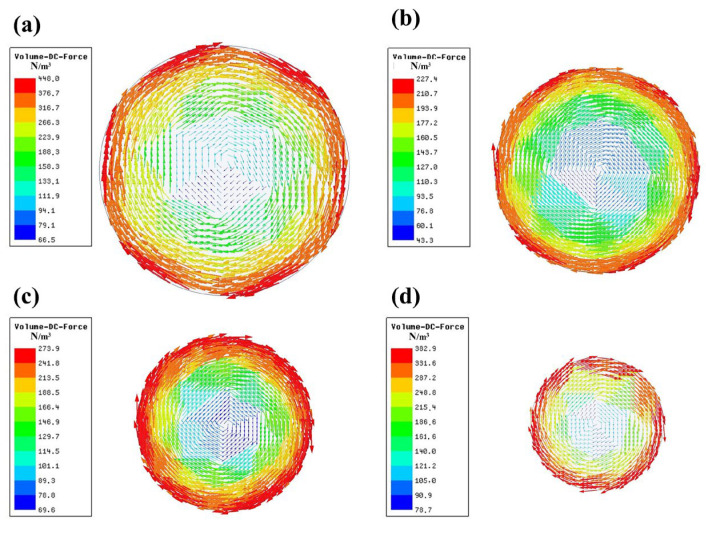
Vector diagram of the electromagnetic force on the strand cross-section in the center of the stirrer: (**a**) Φ650 mm; (**b**) Φ550 mm; (**c**) Φ450 mm; (**d**) Φ350 mm.

When the M-EMS center coincides with the center of the strand, the electromagnetic force distribution on the cross-section of the strand in the center of the stirrer, at the current intensity of 280 A and frequency of 2 Hz, is shown in Figure 11. When the section size is reduced from Φ650 mm to Φ550 mm, the thickness of the mold copper tube is reduced by only 2 mm, but the strand surface is farther away from the coil, so that the magnetic field cannot effectively penetrate the 29 mm thick copper tube after decaying in the air, and thus the maximum electromagnetic force rapidly decays from 440.0 N·m^−3^ to 227.4 N·m^−3^. As the section size continues to be reduced to Φ450 mm and Φ350 mm, the thickness of the copper tube is 25 mm; although the attenuation of the magnetic field in the air is intensified, the copper tube can be also effectively penetrated by the attenuated magnetic field, and the electromagnetic force is greater than that under the section size of Φ550 mm due to the weakened magnetic shielding effect of the copper tube. Although the thickness of the copper tube for section Φ450 mm and Φ350 mm are both 25 mm, when the section size is reduced, the inner diameter of the copper tube is also reduced, resulting in a decrease in volume, which can ultimately increase the electromagnetic force acting on the Φ350 mm strand. Comprehensive analysis shows that under the action of a magnetic field with a certain frequency, the attenuation of the magnetic field in the air that can effectively pass through the copper tube with a certain thickness should be minimized, and thus M-EMS with a section size of Φ650 mm has the highest efficiency in converting electromagnetic energy into kinetic energy.

**Figure 12:**
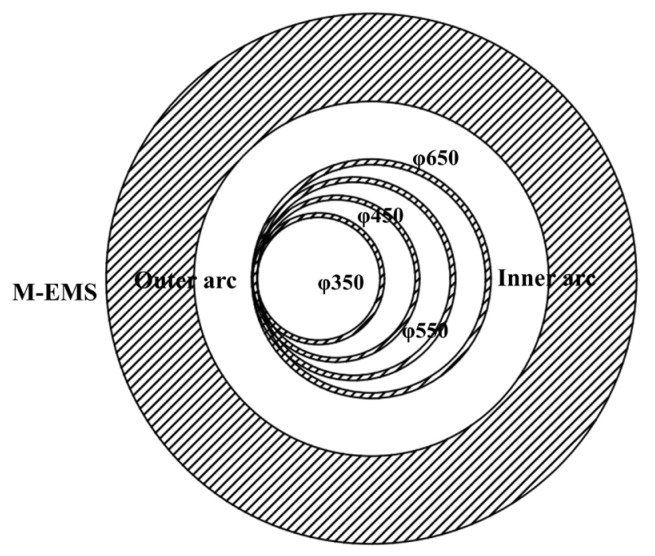
Position of mold copper tube relative to M-EMS for each section size of round blooms.

In the actual production of round blooms in the plant, M-EMS needs to adapt to four types of the strand with section sizes of: Φ650 mm, Φ550 mm, Φ450 mm, and Φ350 mm. The thicknesses of the copper tube for each section are: 31 mm, 29 mm, 25 mm, and 25 mm, respectively. The horizontal installation position of the M-EMS designed according to the maximum section Φ650 mm is fixed with the outer arc as the reference line, which results in a large deviation between the central axis of the strand with a smaller diameter and the central axis of the M-EMS as shown in Figure 12.

**Figure 13:**
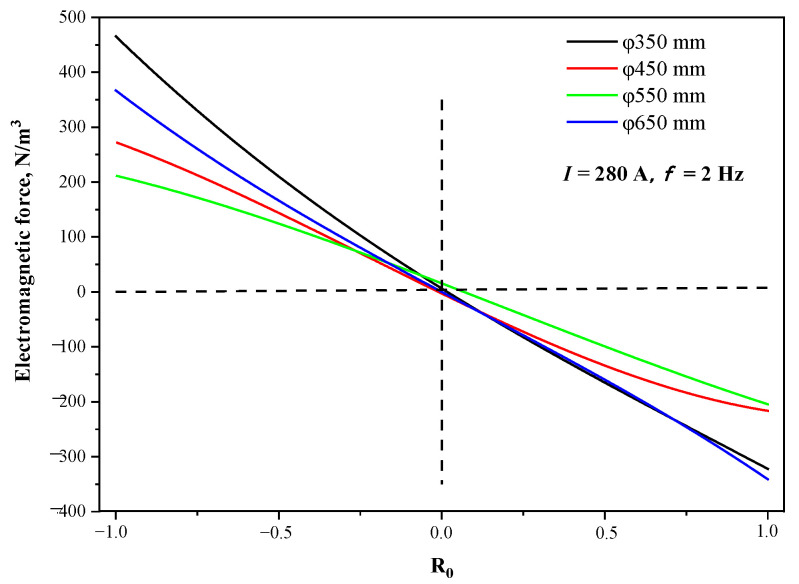
Distribution of electromagnetic force on the radial line of the strand in the center of the stirrer at different sections.

Figure 13 presents the distribution of electromagnetic force on the radial line of the strand in the center of the stirrer, at a current intensity of 280 A and frequency of 2 Hz. The maximum electromagnetic forces under the cross-sections of Φ350 mm, Φ450 mm, Φ550 mm, and Φ650 mm are 465.3 N·m^−3^, 271.6 N·m^−3^, 211.2 N·m^−3^, and 366.9 N·m^−3^, respectively. Because of the combined effect of the magnetic shielding of the copper tube and the attenuation of the magnetic field in the air, the maximum electromagnetic force first decreases rapidly and then increases as the section becomes smaller. In addition, under the eccentric stirring conditions for Φ350 mm, Φ450 mm, and Φ550 mm, the electromagnetic force on the outer arc side of the strand is larger than that on the inner arc side under the proximity effect of the electromagnetic field. The difference in the electromagnetic force values on the inner and outer arc sides is 143.1 N·m^−3^, 55.2 N·m^−3^, and 6.6 N·m^−3^; that is, the electromagnetic force difference increases rapidly as the section size decreases.

## 4. Exploration of Size Effect and Eccentric Stirring for Round Bloom

### 4.1. Effects of Section Size

**Figure 14:**
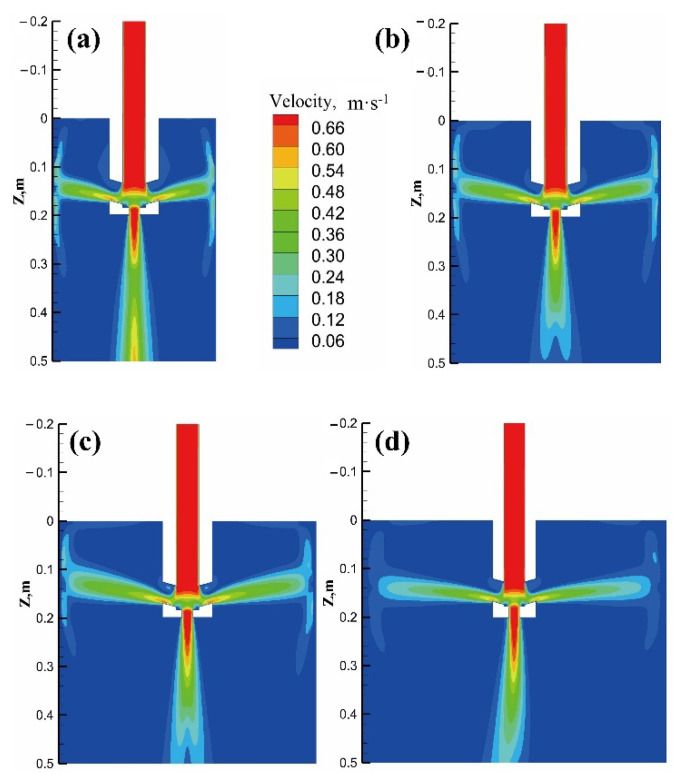
Velocity contour on neutral plane of side port: (**a**) Φ350 mm; (**b**) Φ450 mm; (**c**) Φ550 mm; (**d**) Φ650 mm.

Figure 14 displays the velocity contour on the neutral symmetric plane of the nozzle under different sections. It can be seen from Table 2 that the different casting speeds under different sections result in the inlet velocities in the model being basically the same value, and it can be clearly observed that the velocity of the jet flow from the side port is continuously attenuated under the dissipation effect of the mushy zone, until it impacts the wall and extends to an up and down movement trend. The washing velocity at the solidification front is defined as the value of the velocity on the constant value line with a solid fraction value of 0.9. Extracting the washing velocity information of the solidification front, it can be seen that the maximum washing velocities at the cross-sections of Φ350 mm, Φ450 mm, Φ550 mm, and Φ650 mm are 0.280 m·s^−1^, 0.234 m·s^−1^, 0.213 m·s^−1^, and 0.112 m·s^−1^, respectively; that is, with the increase in the section size, the range of the mushy zone passed by the side-port jet increases, which leads to an increase in the velocity attenuation.

**Figure 15:**
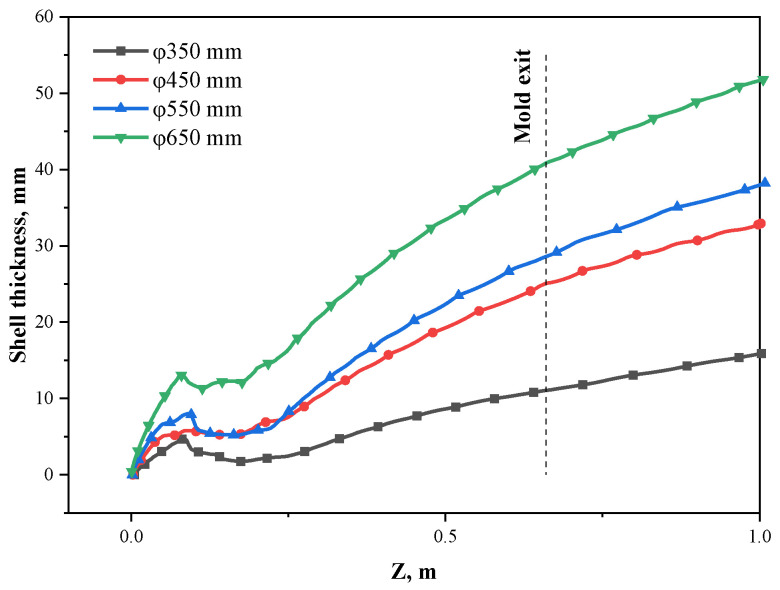
Shell thickness in the impact zone of the side port with different section sizes along the casting direction.

Figure 15 plots the shell thickness along the casting direction under different sections, where the liquid fraction of 0.3 is defined as the location of the solidification shell. It can be seen from the figure that when the section sizes are Φ350 mm, Φ450 mm, Φ550 mm, and Φ650 mm, the shell thickness in the impact zone at the mold exit is 11.1 mm, 25.1 mm, 28.6 mm, and 40.9 mm, respectively. With the increase in the section size of the strand, the shell thickness at the mold exit increases rapidly to offset the influence of the increasing hydrostatic pressure of the molten steel caused by the increase in the section size. According to the research on the safety shell thickness [39], it is estimated that the safety shell thickness at the mold exit with the cross-section sizes of Φ350 mm, Φ450 mm, Φ550 mm, and Φ650 mm is 24.1 mm, 28.4 mm, 32.5 mm, and 36.3 mm, respectively. It can be seen that the shell thickness at the mold exit for Φ350 mm is only 11.1 mm, which is much smaller than the safety thickness, so breakout accidents occur easily, and the Φ450 mm and Φ550 mm sections also have a little risk. Therefore, M-EMS is an essential method in the use of the multi-port nozzle of the round bloom [29]. The effect of electromagnetic stirring on the initial solidified shell thickness in the Φ650 mm section has been investigated in detail, and it has been concluded that 280 A is a relatively better current intensity.

**Figure 16:**
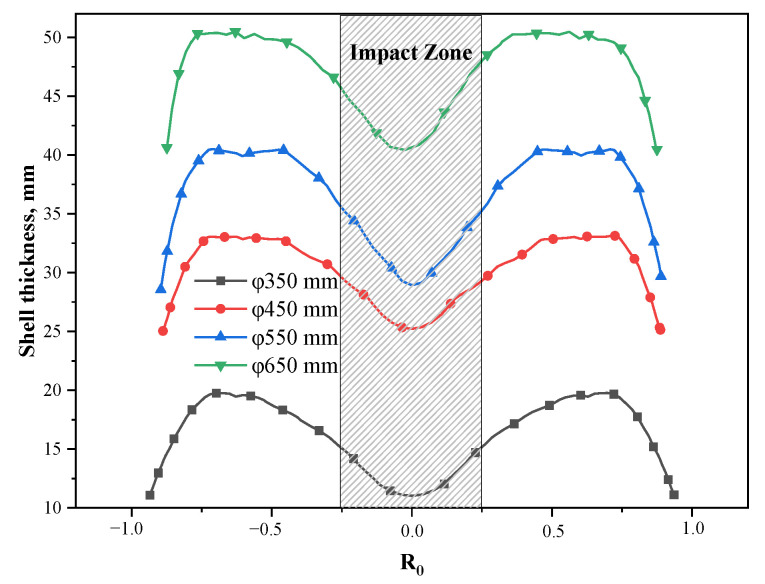
Circumferential shell thickness at the mold exit for different section sizes.

Figure 16 presents the shell thickness in the circumferential direction of the mold exit under different cross-section sizes. The variance of the shell thickness at the mold exit under the section sizes of Φ350 mm, Φ450 mm, Φ550 mm, and Φ650 mm are 2.9 mm, 2.8 mm, 4.0 mm, and 3.3 mm, respectively. In the absence of M-EMS, the uniformity of the shell of the round bloom is poor when the multi-port nozzle is used, which has an adverse effect on its surface quality. In addition, according to the distribution characteristics of the shells under different sizes, that is, when the impact zone of the side port is thin and the other areas are thick, a new type of high-efficiency heat transfer mold copper tube can be designed to solve the safety problem.

### 4.2. Eccentric Stirring

**Figure 17:**
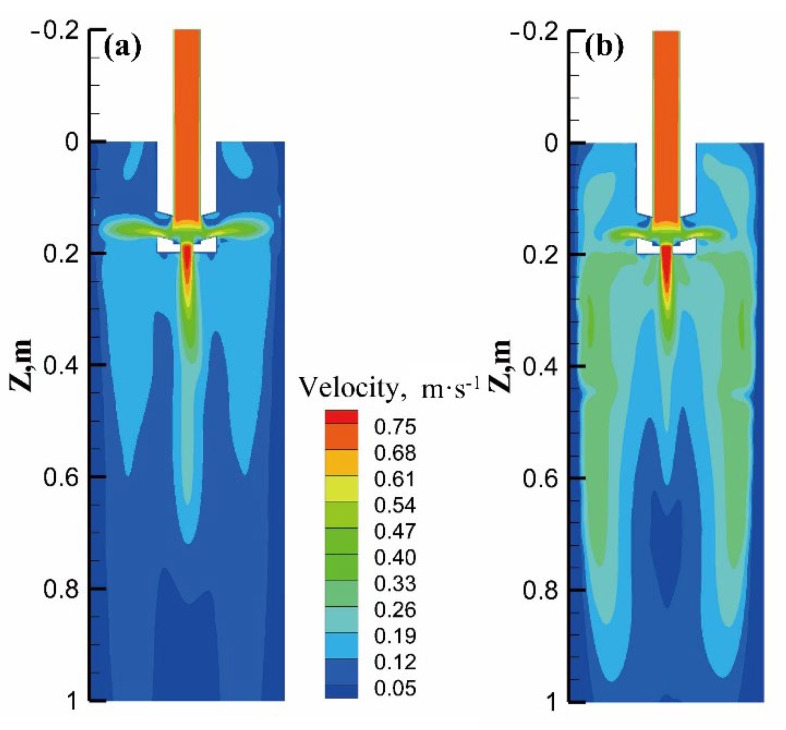
Velocity contour of the neutral plane of the side port under eccentric stirring of Φ350 mm section: (**a**) 280 A; (**b**) 480 A.

Figure 17 shows the velocity contour of the neutral plane of the side port with different current intensities at the section size of Φ350 mm. Combined with Figure 12 and Figure 13a, it can be seen that even the eccentric electromagnetic force can suppress the side-port jet. As the current intensity is increased to 480 A, it is more conducive to reducing the impact of molten steel, showing a flow trend dominated by electromagnetic force. Due to the larger electromagnetic force on the outer arc side, the area of the high-velocity zone on the outer arc side in the lower part of the mold is slightly larger than that on the inner arc side.

**Figure 18:**
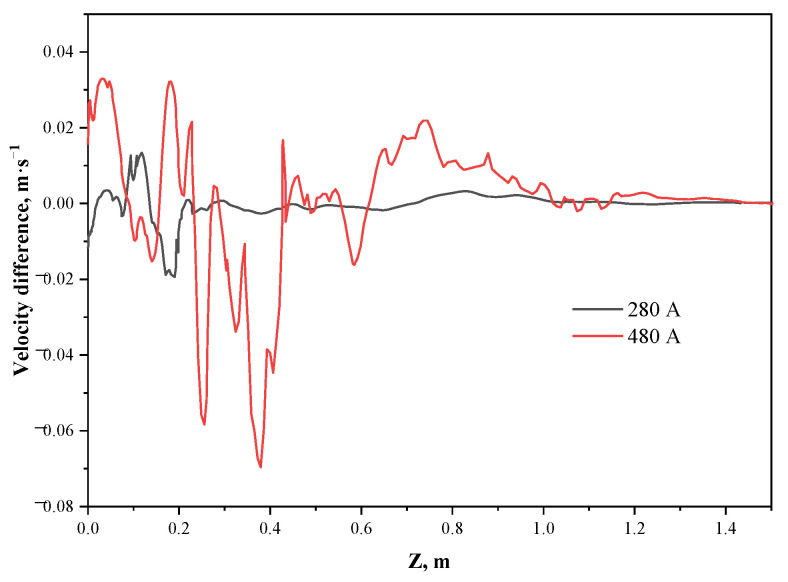
Distribution of the difference in washing velocity at the solidification front on the inner and outer arc sides.

The extracted molten steel washing velocity at the solidification front of the inner and outer arc sides and the velocity difference (inner arc side velocity minus outer arc side velocity) is shown in Figure 18. When the current intensity is 280 A, the effect of the electromagnetic force on the outer arc side of the side port impact zone to suppress the jet is stronger than that on the inner arc side, resulting in larger kinetic energy of the jet retained to the inner arc side. When the current intensity is 480 A, the jets from the side ports of the inner and outer arcs cannot effectively impact the solidification front. At the position of the original impact point, the washing velocity of the outer arc side is dominated by the electromagnetic force, and the washing velocity of the inner arc side near the meniscus and the lower region of the original impact point is higher. The flow in the lower region of the mold is completely dominated by the electromagnetic force, which shows a greater washing velocity on the outer arc side, and the maximum velocity difference can increase from 0.02 m·s^−1^ at 280 A to 0.07 m·s^−1^ at 480 A.

**Figure 19:**
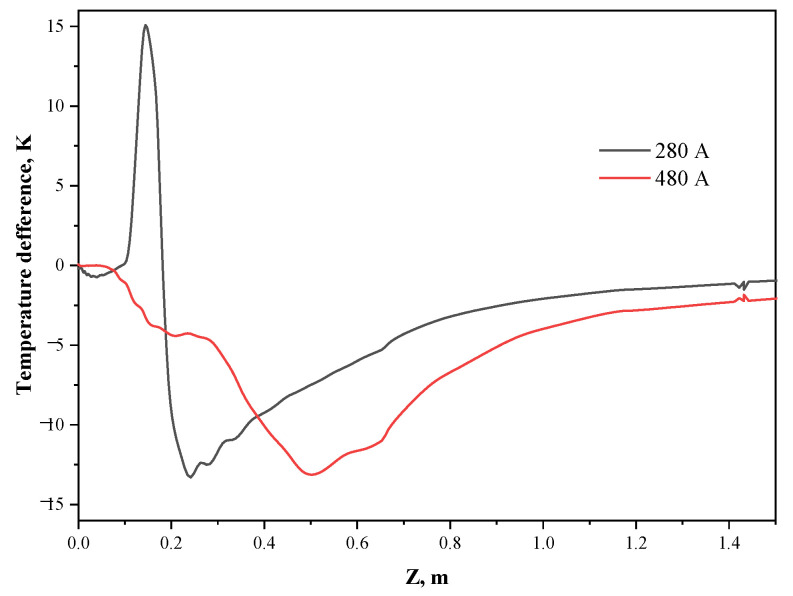
Distribution of surface temperature difference between inner and outer arc sides.

Figure 19 illustrates the distribution of the surface temperature difference (inner arc side temperature minus outer arc side temperature) between the inner and outer arc sides under eccentric stirring. The eccentric electromagnetic force at the current intensity of 280 A causes the heat transfer effect between the high temperature molten steel and the wall in the inner arc region to be higher than that in the outer arc region, and the temperature difference between the inner and outer arc side in this region reaches 15 K under this working condition. In addition, the larger electromagnetic force on the outer arc side of the mold exit area drives the forced heat transfer between the molten steel that has already dissipated a part of the heat in the deeper part of the mold with the initial shell, resulting in higher temperature on the outer arc side than that on the inner arc side under the eccentric stirring. Extracting the shell data, it can be seen that the shell thickness at the mold exit under the current intensity of 480 A is 15.6 mm, and 15.4 mm on the inner and outer arcs, respectively, with a difference of only 0.2 mm. The research [40,41] on the eccentric stirring under the normal straight nozzle of the round bloom shows that the molten steel temperature on the outer arc side is always higher than that on the inner arc side, and the temperature difference first increases and then decreases with the increase in the distance from the meniscus, which leads to the carbon content of the outer arc side being always lower than that of the inner arc side. However, the multi-port nozzle in this study can alleviate the overall difference between the inner and outer arc sides under eccentric stirring to a certain extent, which is beneficial to the homogenized production of round blooms.

## 5. Conclusions

● With the increase in current frequency, the magnetic flux density decreases, but the electromagnetic torque increases and then decreases, and the same optimal stirring frequency exists for the same M-EMS structure for round blooms at any current intensity. The torque value and electromagnetic force both grow as a quadratic function of the current intensity, and the electromagnetic torque, which drives the molten steel flow, can directly characterize the real M-EMS performance.
● The mold copper tube has a significant magnetic shielding effect on the M-EMS. The stirring intensity decreases rapidly as the tube thickness increases, and the optimal stirring frequency decreases as well. In fact, the center of the stirrer deviates from the geometric center of the strand, which results in the eccentric stirring phenomenon. It reveals the magnetic shielding effect of the mold copper tube and the magnetic field loss of the air between the stirrer and the inner and outer arcs of the copper, which lead to the stirring intensity and the eccentric stirring phenomenon.
● When the Φ650 mm SMS-Concast casting machine casting blooms with the section size of Φ350 mm, the washing effect on the upper region of the inner arc side and the lower region of the outer arc side is stronger, so that the temperature of the inner and outer arcs shows alternating differences, and the jet flow from the five-port nozzle can suppress the difference in initial solidification symmetry between the inner and outer arcs of the round bloom caused by eccentric stirring.

## Figures and Tables

**Table 1:** Governing equations of the coupling solidification model.

Governing Equations	Symbols
$\nabla \cdot \vec{B}=0\;\nabla \times \vec{E}=-\frac{\partial \vec{B}}{\partial t}\;\nabla \cdot \vec{D}=q=0\;\nabla \times \vec{H}=\vec{J}+\frac{\partial \vec{D}}{\partial t}=\vec{J}\;\vec{J}=\sigma \vec{E}\;\vec{B}=\mu \vec{H}\;\vec{F_{m}}=\frac{1}{2}Re_{m}\vec{(J}\times \vec{B}*)$	$\vec{B}$, Magnetic flux density [Tesla]
	$\vec{E}$, Electric field strength [V·m^−1^]
	t, Time [s]
	$\vec{D}$, Induced electric field strength [A·m^−2^]
	q, Induced charge density [C·m^−3^]
	$\vec{H}$, Induced magnetic flux density [A·m^−1^]
	$\vec{J}$, Induced current intensity [A·m^−2^]
	σ, Conductivity [S·m^−1^]
	μ, Magnetic permeability [H·m^−1^]
	$\vec{F_{m}}$, Electromagnetic force density [N·m^−3^]
	$Re_{m}$, Real part of the complex number
	$\vec{B}*$, Conjugate complex number of $\vec{B}$
$\nabla \cdot (\rho \vec{v})=0$	ρ, Density of molten steel [kg·m^−3^]
	$\vec{v}$, Velocity of molten steel [m·s^−1^]
$\nabla \cdot (\rho \vec{v}\times \vec{v})=-\nabla p+\nabla \cdot [\mu_{\mathrm{eff}}(\nabla \cdot \vec{v}+\nabla \cdot {\vec{v}}^{T})]+\rho \vec{g}+\vec{F_{m}}+F_{B}+S_{p}\;\mu_{\mathrm{eff}}=\mu_{l}+\mu_{t}=\mu_{l}+\rho f_{\mu }C_{\mu }\frac{\varepsilon^{2}}{k}\;F_{B}=\rho g\beta_{T}(T-T_{\mathrm{ref}})\;S_{P}=\frac{{\left(1-f_{L}\right)}^{2}}{f_{L}{}^{3}+\zeta }A_{\mathrm{mush}}(\vec{v}-{\vec{v}}_{p})\;f_{L}=\left\{\begin{array}{cc} 1 & T\geq T_{L}\\ \frac{T-T_{S}}{T_{L}-T_{S}} & T_{S}<T<T_{L}\\ 0 & T\leq T_{S} \end{array}\right.$	p, Static pressure [Pa]
	$\mu_{\mathrm{eff}}$, Effect viscosity coefficient [kg·m^−1^·s^−1^]
	$\mu_{l}$, Laminar viscosity coefficient [kg·m^−1^·s^−1^]
	$\mu_{t}$, Turbulent viscosity coefficient [kg·m^−1^·s^−1^]
	$\vec{g}$, Acceleration of gravity [m·s^−2^]
	k, Turbulent kinetic energy [m^2^·s^−2^]
	ε, Turbulent energy dissipation rate [m^2^·s^−3^]
	$f_{\mu }$, $C_{\mu }$ from the low Reynolds k-ε model [33]
	$\beta_{T}$, Thermal expansion coefficient
	T, Local temperature [K]
	$T_{\mathrm{ref}}$, Reference temperatures [K]
	$f_{L}$, Liquid volume fraction
	ζ, 0.001
	$A_{\mathrm{mush}}$, 1 × 10^8^
	${\vec{v}}_{p}$, Pull velocity [m·s^−1^]
	$T_{L}$, Liquidus temperature [K]
	$T_{S}$, Solidus temperature [K]
$\nabla \cdot (\rho \vec{v}H)=\nabla \left(k_{eff}\nabla T\right)\;H=h_{ref}+\int_{T_{ref}}^{T}c_{p}dT+f_{l}L\;k_{eff}=k_{l}+\frac{c_{p}\mu_{t}}{{\mathrm{Pr}}_{t}}$	H, Enthalpy [J·kg^−1^]
	$k_{eff}$, Effective thermal conductivity [W·m^−1^·K^−1^]
	$h_{ref}$, Reference enthalpy [J·kg^−1^]
	$c_{p}$, Specific heat [J·kg^−1^·K^−1^]
	L, Latent heat [J·kg^−1^]
	$k_{l}$, Laminar thermal conductivity [W·m^−1^·K^−1^]
	${\mathrm{Pr}}_{t}$, Turbulent Prandtl number [0.85]

**Table 2:** Parameters of the round bloom CC and thermophysical parameters of 42CrMo steel.

Parameter	Value	Parameter	Value
Inside and outside diameter of the nozzle (mm)	45, 90	Molten steel Density (kg·m^−3^)	7020
Effective length of mold (mm)	660	Molten steel Viscosity (kg·m^−1^·s^−1^)	0.0062
Section (mm)	Φ350, Φ450, Φ550, Φ650	Molten steel Specific heat (J·kg^−1^·K^−1^)	750
Thickness of copper (mm)	25, 25, 29, 31	Molten steel Latent heat (J·kg^−1^)	268,000
Casting speed (m·min^−1^)	0.72, 0.42, 0.30, 0.21	Superheat degree (K)	30
Mold cooling water (L·min^−1^)	2800, 4500, 4600, 4600	Solidus temperature (K)	1710
Second cooling ratio (L·kg^−1^)	0.25, 0.18, 0.18, 0.18	Liquidus temperature (K)	1768
Thermal conductivity (W·(m·K)^−1^)	29	/	/

## Data Availability

The data presented in this study are available on request from the corresponding author.
